# A Survey of Image-Based Fault Monitoring in Additive Manufacturing: Recent Developments and Future Directions

**DOI:** 10.3390/s23156821

**Published:** 2023-07-31

**Authors:** Ryanne Gail Kim, Mideth Abisado, Jocelyn Villaverde, Gabriel Avelino Sampedro

**Affiliations:** 1 Research and Development Center, Philippine Coding Camp, 2401 Taft Ave, Malate, Manila 1004, Philippines; ryanne@philippinecoding.com; 2 College of Computing and Information Technologies, National University, Manila 1008, Philippines; mbabisado@national-u.edu.ph; 3 School of Electrical, Electronics and Computer Engineering, Mapúa University, Manila 1002, Philippines; jfvillaverde@mapua.edu.ph; 4 Faculty of Information and Communication Studies, University of the Philippines Open University, Laguna 4031, Philippines; 5 College of Computer Studies, De La Salle University, 2401 Taft Ave, Malate, Manila 1004, Philippines

**Keywords:** additive manufacturing, fault monitoring, machine learning, image-based

## Abstract

Additive manufacturing (AM) has emerged as a transformative technology for various industries, enabling the production of complex and customized parts. However, ensuring the quality and reliability of AM parts remains a critical challenge. Thus, image-based fault monitoring has gained significant attention as an efficient approach for detecting and classifying faults in AM processes. This paper presents a comprehensive survey of image-based fault monitoring in AM, focusing on recent developments and future directions. Specifically, the proponents garnered relevant papers from 2019 to 2023, gathering a total of 53 papers. This paper discusses the essential techniques, methodologies, and algorithms employed in image-based fault monitoring. Furthermore, recent developments are explored such as the use of novel image acquisition techniques, algorithms, and methods. In this paper, insights into future directions are provided, such as the need for more robust image processing algorithms, efficient data acquisition and analysis methods, standardized benchmarks and datasets, and more research in fault monitoring. By addressing these challenges and pursuing future directions, image-based fault monitoring in AM can be enhanced, improving quality control, process optimization, and overall manufacturing reliability.

## 1. Introduction

The use of additive manufacturing (AM) in various manufacturing fields is expanding quickly due to its ability to create parts with complex features. It is a process of developing physical objects from a geometrical representation by fusing materials in discrete planar layers, but non-planar processes also exist [1]. Other terminologies used to describe AM processes include 3D printing (3DP), rapid prototyping (RP), direct digital manufacturing (DDM), rapid manufacturing (RM), and solid freeform fabrication (SFF) [2]. The whole AM process involves 3D computer-aided design (CAD) models to build parts in a layer-wise pattern. Some examples of software that create CAD are SolidWorks, Inventor, Google SketchUp, and Autodesk Revit [3]. Materials used in AM include metals and alloys, ceramics, polymers, composites, smart materials, concrete, and biomaterials [4]. Overall, this process allows individuals to fabricate structures with complex geometric parts that cannot be achieved through traditional methods [5,6,7]. Consequently, it caused a paradigm shift in product design and manufacturing [8].

The 1984 invention of 3D printers by Charles Hull has the potential to revolutionize industries and alter the production line. Over the years, this technology has experienced a phenomenal expansion [9]. AM has applications in electronics, electrochemistry and energy storage, catalysts, thermal management, aerospace, healthcare monitoring, food, sensors, and robotics. For example, AM is used in medical modeling for clinician training and clinician preparation, such as impact planning and pre- and post-operative planning. AM enables surgeons to create operative models for planning and surgical simulations by using imaging datasets as the geometric definitions to model 3D shapes by a variety of software for specific applications. The models are useful for education, but they can also be used to describe risky and difficult surgical procedures to patients and their families [10]. In construction, AM is used to enable automation. Doing so can lower labor for safety reasons, reduce construction onsite, reduce production costs, address sustainability issues, and increase architectural freedom [3]. In general, freedom of design, mass customization, waste minimization, fast prototyping, and the ability to manufacture complex structures are the main benefits of AM [11]. In addition, compared to conventional manufacturing techniques, AM has more controllable process parameters and more vital interaction between the material properties and process parameters [2].

The AM process involves three main phases: (1) the preprocessing phase, (2) the manufacturing phase, and (3) the post-processing phase. Each of these phases is further divided into sub-phases. Several phases, especially post-processing, depend on which AM technology is used. The preprocessing phase has two sub-phases: 3D model creation and data preparation. The 3D model creation sub-phase involves the creation of a 3D model of the object using CAD software or a 3D object scanner. Generally, the data preparation sub-phase converts a CAD model into a format for data handling in AM such as the standard tessellation language (STL) file. This file is then processed by a slicer program, which creates a job file that is saved in the format for the specifically designed machine. Furthermore, the manufacturing phase has two sub-phases: machine setup and building. During the machine setup, the material that will be used is loaded, and the process parameters in the printer are set. Afterward, the printer builds the model by depositing material layer by layer (building sub-phase). Lastly, the post-processing phase identifies the following sub-phases: part removal, support structures, heat treatment, shot-peening, and finishing [12].

**Figure 1:**
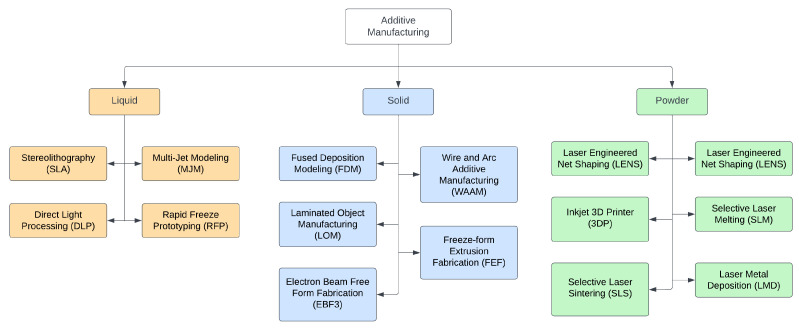
The AM process can be classified in many ways. One of which is the classification based on materials used: liquid, solid, and powder.

The most popular way to classify AM processes is based on the product formation method. According to the American Society for Testing and Materials (ASTM F42), AM processes can be classified into seven categories, namely, material jetting (a drop by drop of build material is selectively deposited), binder jetting (a liquid binding agent is selectively deposited to join powder particles), vat photopolymerization (curing of photo-reactive polymers by the use of a laser, light or ultraviolet), powder bed fusion (uses an electron beam or laser to melt or fuse the material powder), material extrusion (material is extruded through a heated nozzle), energy deposition (similar to material extrusion but the nozzle is not fixed to a specific axis and can move in multiple directions), and sheet lamination (sheets of materials are bonded together to produce a part of the object) [7,13]. Table 1 shows an updated comprehensive overview of the advantages and drawbacks of each process [14,15,16]. Furthermore, these processes are subdivided into a few more related AM technologies. Another way to classify AM technologies is by the type of material used and the medium used for its processing (laser beam, ultraviolet rays, thermal means). Three main types of materials are used in AM: liquid-based, solid-based, and powder-based, as shown in Figure 1 [17]. Other ways to classify AM process are the material preparation, layer generation technique, phase change phenomenon, material type, and application requirements [2].

Despite the advantages of AM, such as design freedom, customization, waste reduction, and the ability to print complex structures, a few disadvantages require additional research and technological development. These particular difficulties include porosity brought on by inadequate material fusion, the anisotropic nature of the materials, and warping due to residual stress brought on by the rapid cooling nature of AM processes. Cracks, delamination, distortion, rough surfaces, lack of fusion, porosity, foreign inclusions, and process instability (keyhole, balling) are specific processing-related faults or defects in AM. These faults are frequently the result of the layer-by-layer material deposition process. In this process, some faults may propagate from one layer to the subsequent layers, causing the entire build to fail [18]. These faults become the cause for high costs and limited applications in large structures and mass production in AM.

**Figure 2:**
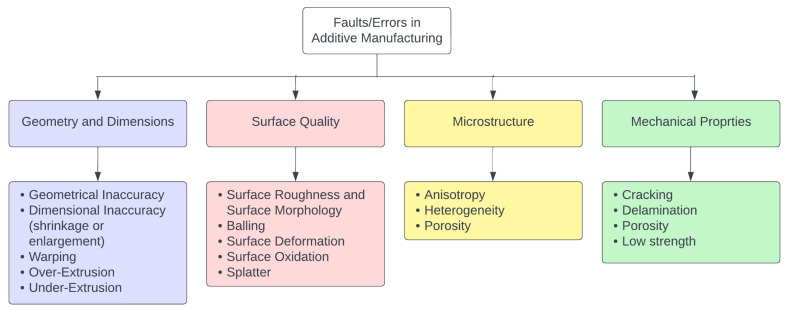
The common errors or faults in AM can be categorized in four ways, according to how it affects the by-product.

The AM output is affected by various essential parameters, including layer thickness, printing speed, printing temperature, and material properties. A detailed understanding of the AM process—from the ability of materials to be processed to the relationship between the process–structure–properties of the AM parts—is crucial to ensure high product quality [5,8,18]. The first step in mitigating faults in AM is understanding the defects and their causes. Listed below are some of the common faults and their definitions. These can be categorized according to how AM affects the by-product, e.g., whether it affects the geometry and dimensions, surface quality, microstructure, or mechanical properties as shown in Figure 2 [19].

- Geometrical Inaccuracy: the deviation of a printed object’s shape or geometry from its intended design due to issues in the printing process, such as incorrect bed leveling, insufficient cooling, or buildup of residual stress [20].
- Warping: occurs when the edges of a printed object curl up or lift from the print bed due to uneven cooling, poor adhesion to the bed, low bed temperature, or residual thermal strain accumulated during the printing [21].
- Balling: occurs when excess material collects and forms a ball or blob on the printed object during the printing process [22].
- Splatter: the unintentional extrusion of material during printing, resulting in excess material or a messy print [23].
- Anisotropy the variation in the mechanical or physical properties of a printed object in different directions, resulting from the layered nature of 3D printing [24].
- Porosity: the presence of voids or holes within a printed object, which can result from incomplete or insufficient printing [25].
- Cracking: occurs when a printed object develops cracks or fractures due to sudden changes in temperature during printing or other issues [25].
- Delamination: the separation or detachment of layers in a printed object due to poor adhesion between layers caused by the improper gap between the nozzle height and print [21].
- Over-Extrusion and Under-Extrusion: Over-extrusion occurs when the 3D printer deposits more material than necessary for each layer of the printed object. On the other hand, under-extrusion occurs when the 3D printer does not deposit enough material for each layer, resulting in incomplete or weak prints. It is caused by too much or a lack of filament flow, respectively [26].

Hence, several types of research have been conducted to monitor faults during printing in the past several years. Fault monitoring in AM is crucial for ensuring the quality and reliability of printed parts. It enables manufacturers to deliver high-quality components, reduce rework and wasted materials costs, optimize printing processes, and drive continuous improvement in the AM industry. By monitoring defects, manufacturers can identify issues early, take corrective actions, and enhance printed parts’ overall quality and integrity, making AM a more viable and trustworthy manufacturing method across various industries. There are two main approaches to monitoring faults during the AM process. The first approach uses analytical models to predict the values of the process parameters. Mathematical and statistical models are applied to the AM process to avoid failures, enhance part quality, and create flawless products to understand its behavior further. A significant computational burden is associated with the current investigations because they all relied on complex simulation and physics-based Finite Element Analysis (FEA). Real-time control requires effective analytical and data-driven models to process large data streams. These issues prompted researchers to consider a second strategy [11].

The second approach is to monitor faults through in-process monitoring. During the AM process, sensors can be incorporated into the printing equipment to monitor critical parameters such as temperature, pressure, laser power, or material flow [18]. The most significant benefit of this method is that it allows for early fault detection and real-time process control. In-process monitoring enables the early detection of defects or anomalies by continuously monitoring key printing process parameters. This permitted immediate intervention prior to the escalation of defects, resulting in improved part quality and a decreased need for costly rework or reprinting. In-process monitoring also provides real-time feedback on the printing process, enabling operators to promptly adjust and control critical parameters. This improves process control and decreases the probability of defects, ensuring consistent part quality throughout the production run. Two subcategories of in-process monitoring exist: image-based monitoring and sensor-based monitoring. The latter entails integrating multiple sensors into the AM system to collect and analyze real-time data on crucial parameters. These sensors provide quantitative measurements of critical printing process variables. The paper focuses on image-based monitoring, which involves capturing visual data during the printing process to analyze and monitor various aspects of the AM process. This technique employs cameras or imaging systems to capture images or videos of the printing area. Using computer vision techniques, the images or videos are then analyzed to extract pertinent information.

In this paper, the researchers conducted an extensive literature review on the topic of “Image-Based Fault Monitoring in AM”. Two major academic databases were utilized: Google Scholar and Mendeley. The main objective of the study was to gather papers from January 2019 to July 2023 specifically related to the application of image-based approaches for fault monitoring in the field of AM. The researchers focused on identifying relevant literature that discussed the use of visual data and image analysis techniques to detect and monitor faults and errors in 3D printers and the AM process. To conduct their search effectively, the authors employed a set of targeted keywords related to the subject matter. The keywords used in the search included: AM, 3D Printers, Fault Monitoring, Defect Monitoring, Anomaly Monitoring, Quality Control, Defect Detection, Anomaly Detection, Image-based, and Vision-based. By using these specific keywords, the researchers aimed to narrow down their search results to papers that addressed the intersection of additive manufacturing and image-based fault monitoring techniques. To perform a more comprehensive search, the researchers utilized Boolean operators such as “AND”, “OR” and “NOT” to combine search terms in metadata effectively. Specifically, statements such as ““Additive Manufacturing” AND (Fault Detection OR Fault Monitoring OR Defect Detection OR Anomaly Detection) AND (Vision-based OR Image-based)” were used. Excluded from the search were papers not related to image-based fault monitoring in AM and papers that did not involve the use of visual data and image analysis techniques. Review papers and papers that excluded the use of any AM processes were excluded.

After the initial search and removal of duplication, a total of 53 papers met the inclusion criteria and were selected to be part of the survey. Table 2 shows the number of publications collected, sorted per year. This table offers insights into the research trends and the volume of work published during each period. Based on the data, it is observed here that 2020 and 2021 offered the most published research. The year 2020 was when image-based fault monitoring in AM gained traction. The decline in 2022 may be attributed to the reduced research productivity due to the COVID-19 pandemic [27]. This paper was written in the first half of 2023 when many papers were still undergoing the publication process; thus, a low publication count was found in this year.

This article is organized as follows: Section 2 introduces the concept of image-based fault monitoring, which will be the basis for the preceding sections. Section 3 discusses and reviews ML algorithms for image-based fault monitoring. Section 4 tackles the insights and trends observed from the reviewed studies. Lastly, Section 5 concludes this survey.

## 2. Image-Based Fault Monitoring

Fault monitoring in AM refers to the systematic process of monitoring and detecting deviations, anomalies, or faults during printing to ensure the printed parts’ quality, integrity, and reliability. It involves continuously monitoring the AM process’s critical parameters, variables, or characteristics and comparing them against predetermined thresholds or expected values. The goal is to identify and address any faults or anomalies that may compromise the final part’s quality or performance. It involves using various techniques, such as in-process monitoring, real-time data analysis, and automated systems, to identify faults or deviations from desired specifications. By monitoring parameters such as temperature, pressure, laser power, material flow, layer deposition, or surface quality, fault monitoring allows for the early detection of defects, material inconsistencies, structural irregularities, or printing errors [77].

**Figure 3:**
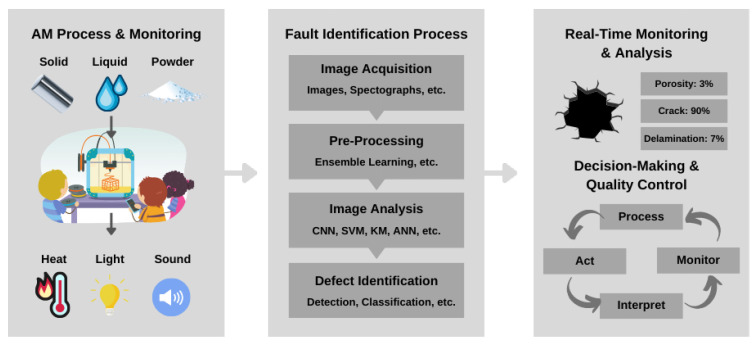
The image-based fault detection process is divided into five main steps: image acquisition, preprocessing, image analysis, defect identification, real-time monitoring and analysis, and decision making and quality control.

One approach to fault monitoring is image-based fault monitoring. During printing, image-based fault monitoring in AM involves capturing visual data using cameras or imaging systems to analyze and detect faults or defects. Using computer vision techniques, this method analyzes captured images or videos and extracts relevant information for fault detection. Image-based monitoring focuses on the visual aspects of the printed part and its printing process, providing valuable information regarding surface quality, layer deposition, feature accuracy, and overall print integrity [78,79]. In image-based fault monitoring, captured images or video frames are examined for irregularities, deviations, or anomalies that may indicate printing defects. Combining multiple techniques, such as using different imaging modalities (e.g., visible light, infrared, X-ray) or employing advanced ML algorithms for automated defect classification, can improve image-based fault detection. The goal is to accurately and efficiently identify defects, ensuring high-quality and reliable AM outcomes. The images can also be used to inspect the surface quality of the printed part, detecting surface defects, warping, or roughness. Additionally, image-based monitoring can detect and analyze specific features or geometries on the printed part to ensure accurate reproduction. By continuously analyzing the visual data in real time, image-based fault monitoring enables operators or quality control personnel to identify and address faults early in the printing process, reducing the risk of producing defective or non-conforming parts [8,80]. Figure 3 illustrates the process of image-based fault monitoring in AM. Each step is further discussed in the following subsections [81].

### 2.1. Image Acquisition

High-resolution cameras or imaging systems capture images of the manufactured parts at various stages of the AM process. These images can be obtained either during the printing process or after its completion. In this step, cameras are strategically positioned to capture the printing area or specific regions of interest. The number and placement of cameras depend on factors such as the size of the printing setup, the complexity of the part, and the desired level of coverage. Multiple cameras may provide different views or angles for comprehensive monitoring. The camera settings and parameters are configured to optimize image acquisition. This includes adjusting parameters such as exposure time, aperture, ISO sensitivity, white balance, focus, and frame rate. These settings are adjusted to ensure clear and correctly exposed images or video frames [82].

The images are captured at appropriate intervals based on the specific requirements of the AM process. Factors including layer deposition time, cooling periods, or critical stages of the printing process can determine this. The camera is also calibrated to ensure accurate and reliable measurements from the captured images. This involves determining the camera’s intrinsic parameters, such as focal length, lens distortion, and pixel size. It helps correct geometric distortions and ensure accurate measurements in subsequent image analysis steps. Two types of cameras are used in image acquisition, namely, optical and thermal.

#### 2.1.1. Optical Camera

Optical cameras capture images within the visible light spectrum. These cameras function similarly to conventional cameras and can capture images with high resolution and color accuracy. Versatile and widely used in AM for monitoring the printing process and detecting visible flaws or inconsistencies, they are adaptable and versatile. Optical cameras can provide visual data on the printed object, such as layer deposition, surface quality, and geometry. They can capture images of each layer or specific regions of interest, enabling real-time monitoring and detection of flaws such as surface roughness, delamination, warping, or missing layers. Optical cameras are beneficial for detecting visible anomalies that may compromise the printed part’s structural integrity or final quality.

Other notable types of optical cameras are high-speed cameras, charge-coupled device (CCD) cameras, and complementary metal oxide semiconductor (CMOS) cameras. High-speed cameras capture images at a rapid frame rate, allowing for the detection of fast dynamic events during the printing process. These cameras can capture fine details and be used to monitor the deposition of each layer or detect defects in real time. CCD cameras offer several advantages regarding image quality, sensitivity, and dynamic range. They can capture high-resolution images with low noise, making them suitable for detailed imaging and analysis. CCD cameras are often used in scientific and industrial applications where image quality and accuracy are crucial [83]. CMOS cameras, on the other hand, have become popular alternatives to CCD cameras due to their lower power consumption, faster readout speeds, and cost-effectiveness. CMOS cameras are also widely used in AM and often provide comparable image quality [82].

#### 2.1.2. Thermographic Camera

Thermographic cameras, also called infrared cameras, capture images based on objects’ heat or thermal radiation. These cameras operate in the non-visible infrared spectrum and are sensitive to temperature differences. By detecting variations in thermal patterns, thermographic cameras can identify areas of heat generation or dissipation, enabling the detection of thermal anomalies during the AM process [84]. Thermographic cameras help monitor AM-related issues, such as overheating, cooling inconsistencies, or thermal gradients. These anomalies may indicate faults such as improper material fusion, insufficient cooling, or insufficient energy input. By detecting these thermal irregularities, thermographic cameras can help ensure the integrity and quality of the printed part.

### 2.2. Preprocessing

The acquired images may undergo preprocessing steps to enhance the quality and extract relevant information [85]. Preprocessing aims to improve the clarity and consistency of the images for subsequent analysis. This includes steps such as image cleaning to remove unwanted artifacts, image filtering to reduce noise, contrast enhancement to improve the visibility of details, image registration to align multiple views, calibration to correct geometric distortions, image resampling for specific requirements, illumination correction to equalize lighting conditions, and image segmentation to isolate relevant regions or objects. These preprocessing steps ensure the captured images are high quality, free from disturbances, and adequately prepared for subsequent fault detection and analysis. This enables accurate and reliable identification of faults or defects in the AM process.

### 2.3. Image Analysis

Image analysis techniques are then applied to examine the preprocessed images. This involves extracting meaningful features from the images that can be used to identify defects. Different methods may be employed, including:

- Image segmentation: This process involves partitioning the image into meaningful regions or objects. It separates the defects from the background or surrounding structures, making them easier to analyze separately [86].
- Feature extraction: Relevant features are extracted from the segmented regions or the entire image. These features can include geometric characteristics (e.g., shape, size, or aspect ratio), texture patterns, intensity profiles, or statistical measures [87,88].
- Classification: ML algorithms or pattern recognition techniques can classify the extracted features and distinguish between normal and defective parts. This may involve training a classifier on labeled data, where the defects are identified and associated with specific feature patterns [82].

### 2.4. Defect Identification

After classifying the features, the presence and type of defects can be determined. Surface irregularities, cracks, voids, porosity, warping, and other flaws may constitute defects. The analysis can provide information regarding the defects’ location, size, severity, and nature. Listed below are the steps involved in the process of identifying defects:

1. Fault localization: The first step in defect identification is to determine the precise location of the detected fault within the captured images or video frames. This involves mapping the identified features or anomalies to the corresponding regions of the AM process. Localization helps pinpoint the specific area where the fault or defect has occurred [82].
2. Categorization and classification: Once the fault is localized, it is categorized and classified based on its nature and characteristics. This step involves assigning a specific category or type to the detected fault, such as missing layers, surface irregularities, dimensional deviations, or structural defects. Classification helps understand the fault’s nature and facilitates subsequent analysis and decision making [82].
3. Severity assessment: The severity of the detected fault is assessed to determine its impact on the quality and functionality of the printed part. This involves evaluating the extent of the defect, its potential to compromise structural integrity, or its effect on critical dimensions or functional properties. Severity assessment helps prioritize the detected faults and guides subsequent actions for mitigation or correction.
4. Reference comparison: In some cases, a reference comparison is performed to assess the detected fault against a known reference standard. This involves comparing the features or characteristics of the faulty part with those of a defect-free reference part or an ideal model. Reference comparison provides a basis for evaluating deviations or abnormalities and determining the acceptability of the printed part.

### 2.5. Real-Time Monitoring and Decision Making

The defect detection and classification process is performed in real time as new images are acquired during the AM process. The system continuously monitors the images and provides immediate feedback on defects or anomalies. Real-time monitoring allows for timely intervention and adjustment of the manufacturing process to prevent further defects. The system generates alerts or notifications to inform the operators or control systems based on the detected defects or anomalies [89]. The alerts can trigger actions such as pausing the process, adjusting parameters, or initiating corrective measures. The decision-making process relies on predefined criteria or quality standards to determine the acceptability of the manufactured part. Data collected, including images, extracted features, and defect classifications, can be logged for further analysis and quality control. These data can be used for process optimization, defect trend analysis, and continuous improvement of the AM process.

## 3. Machine Learning Algorithms

According to [90], ML is a set of methodologies and algorithms capable of extracting knowledge from data, and they continuously improve their capabilities by learning from experience. ML is a subfield of artificial intelligence (AI) that focuses on developing algorithms and models that enable computers and systems to learn from data and make predictions or decisions without being explicitly programmed. It involves constructing and analyzing mathematical models and algorithms that allow computers to learn and improve from experience, iteratively adapting their performance based on available data. In other words, it involves designing and implementing algorithms that can automatically learn and improve based on experience or data without explicit instructions for each specific task. The fundamental premise of ML is to enable computers to automatically discover patterns or relationships in data and make intelligent predictions or decisions. Training the ML model on a large dataset enables it to recognize patterns, extract pertinent features, and generalize from the provided examples. The model learns from the data by iteratively adjusting its internal parameters or structure to reduce errors and enhance performance on the given task. ML techniques are broadly classified as supervised, unsupervised, semi-supervised, and reinforcement learning [91].

- Supervised learning: In supervised learning, the model is trained on labeled data, where each example is associated with a corresponding target or output value. The model learns to map inputs to outputs based on the provided labeled examples, enabling it to make predictions on new, unseen data [92].
- Unsupervised learning: Unsupervised learning involves training the model on unlabeled data. The goal is to uncover hidden patterns or structures within the data without explicit guidance. Clustering, dimensionality reduction, and anomaly detection are common tasks in unsupervised learning [92].
- Semi-supervised learning: Semi-supervised learning combines supervised and unsupervised learning. It uses a small amount of labeled data and a larger amount of unlabeled data to improve the learning process. This can be useful when obtaining labeled data is expensive or time-consuming [92,93].
- Reinforcement learning: Reinforcement learning focuses on training an agent to interact with an environment and learn optimal actions to maximize a reward signal. The agent explores the environment, receives feedback through rewards or penalties, and adjusts actions to achieve the desired goal [94,95].

**Figure 4:**
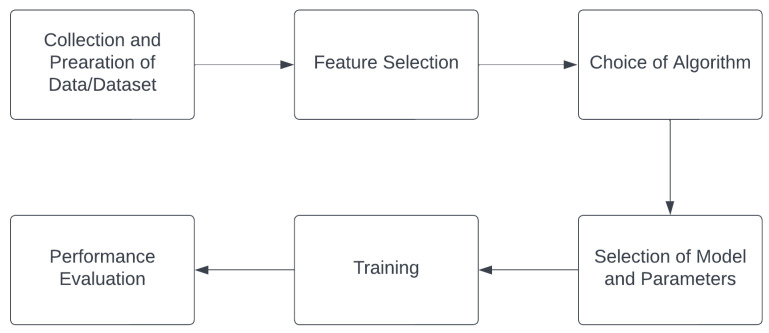
The generic ML model consists of six components: collection and preparation of data/dataset, feature selection, choice of algorithm, selection of model and parameters, training, and performance evaluation.

According to [96], the generic model of ML consists of six components, independent of the algorithm adopted. Figure 4 shows the primary components of ML with each component having a specific task to accomplish:

1. Collection and preparation of data: This involves the gathering of relevant data that will be used to train and test the model. The data should represent the problem domain accurately and have sufficient quantity and quality. Data collection may involve the use of sensors, web scraping, database queries, surveys, or other means. After gathering the data, they need to be preprocessed and prepared for training. Data preprocessing includes tasks such as handling missing values, normalization, feature scaling, handling outliers, and converting categorical variables into numerical representations.
2. Feature selection: In this step, the most relevant and informative features are chosen from the prepared dataset. Feature selection is essential because irrelevant or redundant features can lead to overfitting and increase the model’s complexity without improving its performance. Various techniques such as correlation analysis, forward/backward selection, and feature importance from models can be used for feature selection.
3. Choice of algorithm: Selecting an appropriate machine learning algorithm is critical to the success of the model. The choice of algorithm depends on the type of problem (e.g., classification, regression, clustering), the nature of the data (e.g., structured or unstructured), the size of the dataset, and other factors. Some common algorithms used in AM are discussed in the latter part of the paper.
4. Selection of models and parameters: Once the algorithm is chosen, the next step is to select the specific model and parameters. Most machine learning algorithms require some initial manual intervention for setting the most appropriate values of various parameters.
5. Training: Training is the process of feeding the prepared data into the selected machine learning model and adjusting its parameters based on the input to improve its performance. During training, the model learns patterns and relationships in the data to make predictions or decisions. The model is trained on a portion of the data known as the training set, while the remaining data are reserved for evaluation (testing).
6. Performance evaluation: After the model is trained, its performance needs to be evaluated to determine how well it generalizes to new, unseen data. Performance evaluation is performed using metrics suitable for the specific problem type. For instance, accuracy, precision, recall, and F1-score are common metrics for classification problems, while mean squared error (MSE) and R-squared are used for regression problems. Performance evaluation helps in assessing the model’s effectiveness and identifying potential issues such as overfitting or underfitting.

In AM, ML can be used for the real-time detection and monitoring of faults. ML algorithms can analyze real-time images or video streams captured during printing. By training models on a large dataset of defect images, the algorithms can learn to identify and classify defects, such as surface roughness, voids, cracks, or warping. Additionally, these algorithms can analyze sensor data such as temperature, humidity, gas emissions, and vibrations in real time to identify patterns or anomalies associated with defect formation. Other applications are 3D printing design, process optimization, and security [8,18]. The most common ML algorithms used in image-based fault detection in AM are the following:

### 3.1. Neural Network (NN)

**Figure 5:**
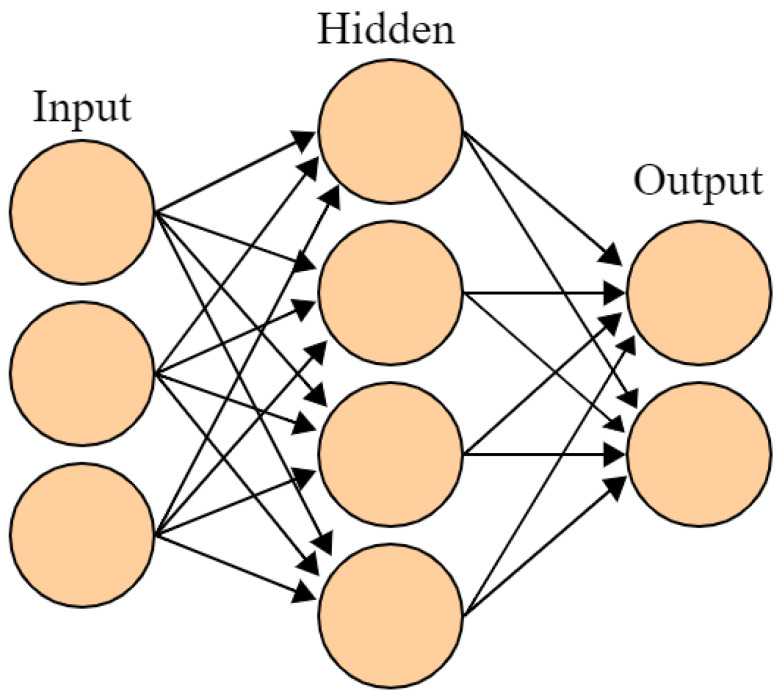
The NN algorithm consists of three types of layers: the input layer, hidden layers, and output layer [100].

The NN algorithm, also known as Artificial Neural Network (ANN), is a class of machine learning techniques that emulates the functionality of interconnected neurons in the human brain. Its architecture consists of three fundamental types of layers: the input layer, hidden layers, and output layer as shown in Figure 5 [97]. Each layer comprises interconnected nodes or neurons, which borrow the idea from neurological sciences. These neurons receive input data, perform computations on that data, and produce outputs that are passed to subsequent layers. The connections between neurons are associated with weights, which represent the magnitudes of influence one neuron has on another within adjacent layers. The weights play a critical role in determining the behavior and effectiveness of the neural network. The training process of a neural network involves iteratively adjusting these weights to minimize the loss function, which measures the discrepancy between the predicted outputs and the actual outputs in the training data. The objective is to find the optimal configuration of weights that allows the neural network to make accurate predictions on unseen data. To achieve this, a variety of optimization techniques are employed, with one of the most famous and widely used being backpropagation. Backpropagation is a mathematical algorithm that uses the chain rule from calculus to compute the gradients of the loss function with respect to each weight in the network. These gradients indicate the direction and magnitude of the change required to minimize the loss. By iteratively updating the weights in the opposite direction of the gradients, the neural network learns to adjust its connections in a way that reduces prediction errors. This process is typically performed over multiple iterations, referred to as epochs, until the neural network converges to a state where the loss is minimized [98]. Once the training phase is completed successfully, the neural network is capable of inferring outputs based on previously unseen inputs. This ability to generalize from the training data to unseen data is one of the key strengths of neural networks, making them highly effective in tasks such as image recognition, natural language processing, speech recognition, and more. Over the decades of its development, researchers have proposed various specific types of neural networks to address different tasks and challenges. Some of these include feedforward neural networks, convolutional neural networks (CNNs) for image processing, recurrent neural networks (RNNs) for sequential data, and generative adversarial networks (GANs) for generating realistic data [99].

### 3.2. Convolutional Neural Network (CNN)

A convolutional neural network (CNN) is a specialized type of feedforward neural network capable of automatically extracting features from data using convolutional structures. Although CNNs have been successfully employed for image-based tasks such as detection, segmentation, and recognition since the early 2000s, their prominence soared following the introduction of the AlexNet architecture during the ImageNet competition in 2012 [101,102]. This event marked a turning point in the adoption and popularity of CNNs in the field of computer vision.

In a CNN, each artificial neuron corresponds to a biological neuron, and the CNN kernels represent receptors that can respond to various features, akin to how neurons in the visual cortex are sensitive to specific patterns [103]. CNNs employ activation functions, such as the rectified linear unit (ReLU), to simulate the thresholding mechanism of neurons, ensuring that only electric signals exceeding a certain threshold are transmitted to the next layer. The training process of a CNN involves the use of loss functions and optimizers to teach the network to learn the desired patterns and relationships from the data. The fundamental components of a CNN model include the following. These layers work together to extract hierarchical representations from input data and perform the final classification or regression task [6].

1. Convolutional layers: Convolutional layers employ a set of learnable filters, also known as a filter bank, to scan the input data in a localized manner, capturing local features and patterns [102]. The filters, represented by weights, are trained during the learning process to detect specific features relevant to the task at hand, such as edges, corners, or textures. This localized scanning and feature extraction process is repeated across the input data, generating feature maps that highlight relevant patterns.
2. Pooling layers: Following the convolutional layers, pooling layers are utilized to reduce the spatial dimensions of the feature maps while retaining the most salient information. Pooling involves applying aggregation functions (e.g., max pooling or average pooling) within localized regions of the feature maps, effectively reducing their size and computational complexity. This downsampling step helps make the network more robust to small variations in the input data and reduces the risk of overfitting.
3. Fully connected layers: The output of the convolutional and pooling layers is then fed into fully connected layers. In these layers, each neuron is connected to every neuron from the previous layer, forming a dense connectivity pattern. These fully connected layers are responsible for making the final decision or prediction based on the extracted features. For image classification tasks, the last fully connected layer often produces the probabilities or scores for each class, and the highest-scoring class is considered the predicted label.

CNNs have proven highly effective in computer vision tasks, including image classification, object detection, and segmentation, owing to their ability to automatically learn intricate features and their advantage of local connections, weight sharing, and downsampling dimension reduction. The combination of these appealing characteristics makes CNNs a prominent algorithm in the field of deep learning, revolutionizing image-based machine learning and artificial intelligence research [104].

### 3.3. Support Vector Machine (SVM)

Support Vector Machine (SVM) is primarily used for categorization and binary classification tasks. It operates on the concept of calculating margins, aiming to find an optimal decision boundary, represented as a hyperplane, that effectively separates different groups of data [105]. The main objective of SVM is to maximize the margin between the decision boundary and the closest data points of each class, known as support vectors. This ensures that the classification error is minimized and enhances the algorithm’s ability to generalize well to new, unseen data. To achieve this, SVM selects the hyperplane that maintains the maximum distance between the support vectors of different classes. These support vectors play a crucial role in defining the decision boundary and are instrumental in constructing the classification model [106]. Given a labeled dataset with samples from different classes, SVM’s architecture involves finding the optimal hyperplane that best separates the data points. This process ensures that the margin between the decision boundary and the labeled classes is maximized, resulting in an effective reduction in the classification error. During the classification phase, when new data are presented, SVM assigns the data point to the class corresponding to the side of the decision boundary on which it lies. The ability of SVM to draw a line or hyperplane to separate data points makes it highly suitable for binary classification problems. In AM, this algorithm is successfully used for detecting part defects, diagnosing faults in 3D printers, and generating process maps [107].

### 3.4. K-Nearest Neighbors (KNN)

K-nearest neighbors (KNN) is a non-parametric and instance-based machine learning algorithm used for classification tasks. The algorithm operates by identifying the K most similar data points (neighbors) from the labeled dataset to a given query example [108]. These neighbors are then utilized to determine the class label of the query through simple majority voting or distance-weighted voting. During the training phase, the labeled dataset is fed into the KNN classifier or learner. The training data consist of input samples along with their corresponding class labels. This step enables the algorithm to learn the underlying patterns and relationships in the data. In the classification phase, when a new query example is presented for prediction, the KNN algorithm searches for the K most similar data points from the training set. The similarity between data points is typically measured using distance metrics, such as Euclidean distance or Manhattan distance. Once the K-nearest neighbors are identified, the class label for the query example is determined based on a voting scheme. In the case of simple majority voting, the class that appears most frequently among the K neighbors is assigned to the query example. This means that the class label with the highest number of occurrences among the neighbors becomes the predicted class for the query. Alternatively, KNN can use distance-weighted voting, where the contribution of each neighbor to the prediction is weighted based on its proximity to the query example. Closer neighbors have a stronger influence on the prediction, while more distant neighbors have a weaker impact.

### 3.5. Decision Tree (DT)

A decision tree (DT) is a hierarchical, tree-like structure used for classification purposes. It organizes and categorizes data by recursively sorting attributes based on their values and grouping them together to make decisions. The decision tree consists of nodes and branches, where each node represents an attribute that requires classification, and each branch represents a specific value taken by that attribute. The process of constructing the tree involves partitioning the data at each node based on the attribute values, leading to the formation of distinct branches corresponding to different attribute value ranges. Ultimately, the leaves of the DT represent the classification outcomes or the predicted classes for the data instances based on the attribute conditions defined by the path from the root to the respective leaf [109].

### 3.6. Random Forest (RF)

Random Forest (RF) is an ensemble learning method used for classification and regression tasks. It consists of a large number of individual decision trees that operate as an ensemble. In this method, each decision tree in the forest independently classifies the input data, and the class with the most votes from the individual trees becomes the final output of the model. During the training phase, the Random Forest algorithm builds multiple decision trees using a technique called bootstrapped aggregation (or bagging). It randomly samples the training data with replacements to create different subsets of the dataset for each tree. This process introduces diversity among the individual trees, as they are trained on different subsets of the data. Each decision tree in the Random Forest independently makes predictions based on the features of the input data. The decision-making process involves splitting the data at each node based on the features that best separate the samples belonging to different classes or, in the case of regression tasks, predict the target values effectively. Once all the decision trees are trained, during the prediction phase, the input data are passed through each tree. Each tree in the ensemble classifies the data independently, and the class label with the highest frequency among the individual trees is chosen as the final output of the RF model [110].

### 3.7. Naive Bayes (NB)

Naive Bayes (NB) is a probabilistic machine learning algorithm primarily used for clustering and classification tasks. It is based on the concept of Bayesian networks, which are graphical models representing the probability relationships among a set of random variables. These graphical models combine graph theoretic approaches with probability theory. In a Bayesian network, the relationships between variables are represented using directed acyclic graphs. Each node in the graph corresponds to a random variable, and the connecting arcs between nodes indicate the probabilistic dependencies between the variables. The conditional probability distribution is used in the underlying architecture of Naive Bayes. The Naive Bayes algorithm assumes that the features (variables) are conditionally independent given the class label. This is known as the “naive” assumption, which simplifies the calculation of probabilities. Despite this simplification, Naive Bayes has shown effective performance in many real-world applications. During the training phase, Naive Bayes estimates the conditional probabilities of each feature given each class label from the labeled training data. It computes the likelihood of each feature occurring in each class. During classification, when a new instance with an unobserved class label is given, the Naive Bayes algorithm utilizes the computed conditional probabilities to calculate the posterior probability of each class. The class with the highest posterior probability is assigned to the new instance as the predicted class label [111].

## 4. Discussion

This section provides a concise summary of the main findings and key points of the research garnered. The advancements and current state of image-based fault monitoring in AM are highlighted here. Furthermore, the recent developments and future directions are discussed in this section.

### 4.1. Summary of Findings

Image-based monitoring is a promising approach for improving the quality and reliability of AM processes. It can be used to detect a variety of defects, including porosity, cracks, surface roughness, and dimensional inaccuracy. This subsection gives an overview of the different types of image-based fault monitoring methods developed for AM. Table 3 shows the research summary collected in this paper. This table is categorized according to the year, AM process, camera used, ML algorithm, and errors detected. These details were included to consider the variations in equipment, approaches, and techniques that may be introduced in future experimentation. “AM process” shows the type of AM process or 3D printer used in the research as part of what was previously discussed in this paper. “Camera Used” includes the type of camera used and its model (if provided). "Proposed Algorithm" and “Accuracy” present the novel or optimized algorithm used in the research for image classification for defect detection and its accuracy. Accuracy is a common indicator of accuracy, along with recall, precision, and F1-score. The most common definition of accuracy is $Accuracy=\frac{TP+TN}{TP+TN+FP+FN}$ wherein TP represents the number of true positive predictions, TN represents the number of true negative predictions, FP represents the number of false positive predictions, and FN represents the number of false negative predictions [112]. Finally, “Errors Detected” lists the error/s detected or mitigated in the experiment.

The types of cameras usually used for image acquisition fall into two categories: optical (digital) cameras and thermographic (thermal or infrared) cameras, as discussed in the previous sections. There are other vision-based methods such as 3D scanners, area-scan hyperspectral cameras, 3D scanning coordinate-measuring machines, and multispectral photodetector sensors. Three-dimensional scanners capture geometric data from the surface of an object, generating a three-dimensional representation of the scanned object. They are utilized for surface defect detection, layer inspection, or dimensional analysis [57]. The area-scan hyperspectral camera is an advanced imaging device that combines the capabilities of an area-scan camera with hyperspectral imaging technology. It captures images across multiple spectral bands, allowing for detailed analysis and characterization of materials based on their spectral properties. It was used by [58] to inspect the surface roughness of printed materials. A 3D scanning coordinate-measuring machine (CMM) is a specialized device that combines the capabilities of a traditional CMM with 3D scanning technology. It is used for precise dimensional measurement and surface scanning of objects, such as in [28]. The multispectral photodetector sensor is an imaging device that captures images across multiple spectral bands or wavelengths. It was used by [45] to estimate porosity in real-time in an L-PBF process.

The most commonly used algorithm in image-based fault detection in AM is CNN. CNNs have demonstrated remarkable performance in identifying various types of faults, including voids, porosity, cracks, and surface irregularities. Specifically, [21,26,30,31,32,35,37,39,40,41,43,44,46,47,50,52,55,58,60,61,71,72] used CNN or a CNN-based algorithm. CNN is commonly used in this application due to its ability to learn spatial hierarchies of features. This is important for fault detection in AM because many faults can be identified by the presence of specific patterns of features in the images. Additionally, it is able to learn invariant features. Lastly, it is able to learn from a large amount of data. CNNs are able to learn from these data to improve their accuracy at identifying faults. In addition to these reasons, CNNs are also relatively easy to train and deploy. For instance, in the SLM process, Ref. [32] shows that CNN has an accuracy of 99.4%. In the aforementioned research, deep CNN (DCNN) is able to automatically extract multi-level image features and discover the embedded patterns that are most relevant to the given problem via supervised learning. A bi-stream DCNN structure is created to analyze SLM part slices and powder layer images for defects brought on by unfavorable SLM process conditions. The next powder layer’s surface pattern may be affected by the part slices’ surface pattern deviation brought on by the varying process conditions. In turn, the irregularity in the powder layer may affect the surface pattern of the next part slice. The bi-stream DCNN can combine the patterns found in both layers of images to jointly classify the defects resulting from non-conformities in the SLM process. In the L-PBF process, CNN also shows a favorable accuracy of 96.8%. In this work, in situ off-axis thermographic imaging serves as the foundation for the ML architecture. For automatic defect detection, a CNN was trained and assessed. Specifically, their network architecture consists of three blocks of CNN and batch-normalization layers as the depthwise separable convolutions were used in blocks 2 and 3.

Another algorithm used in AM is SVM, which was used in papers [34,51,56,59,66,74]. SVM is also commonly used in this application because it is able to learn non-linear relationships between features. It is also able to handle high-dimensional data and is relatively easy to train and deploy. However, there are some limitations to using SVMs since they can be computationally expensive to train. In addition, they can be sensitive to the choice of hyperparameters. Ref. [66] shows that SVM reaches an accuracy of 99.8%. In this study, the surface points of the deposition region were first extracted from the measured 3D surface profiles in the analysis frame, and after non-linear normalization, they were transformed into 2D topography images. The topography image’s pixels were divided into categories for normal and defective pixels using SVM in order to identify the defects. In [74], six supervised machine learning models (with SVM as one of them) were optimized and deployed to classify or predict performance for each experiment (single strand and strand fusion) based on in situ IR thermocouple sensing features. It shows that SVM has the highest performance of >85% in terms of predicting the fusion ratio of various interstrand gaps in bio-AM materials.

In summary, these papers have collectively demonstrated the efficacy of image-based fault detection techniques in AM. The choice of image acquisition methods has played a crucial role in capturing the necessary information for fault detection. Furthermore, the papers explored how CNNs have emerged as a prominent approach, showcasing their ability to accurately identify various types of faults such as voids, porosity, cracks, and surface irregularities. It is worth noting that while CNNs are commonly used, other machine learning algorithms such as SVM and KNN can also be effective depending on the specific requirements and characteristics of the dataset. Moving beyond the existing research, recent developments in this field have focused on tackling remaining challenges and exploring novel approaches. These recent advancements pave the way for further advancements in image-based fault detection in AM, indicating exciting directions for future research and industry applications.

### 4.2. Recent Developments

Significant advancements have been made in image-based fault monitoring in AM in recent years. The integration of imaging techniques, ML algorithms, and sensor technologies has revolutionized the detection and correction of flaws and defects during AM. This section overviews the most recent advancements in image-based fault monitoring in AM, highlighting the most significant hardware and software innovations and developments. These advancements have brought us closer to real-time, automated, and dependable fault detection, paving the way for improved quality control and process optimization.

#### 4.2.1. Image Acquisition

Recent studies have shown that digital and IR cameras are still the dominant image acquisition method; however, there is a trend in using multi-modal cameras and other vision sensors such as digital microscopes and laser profilometer sensors. When only one camera is used for sensing purposes during an experiment, the layer height is the primary standard parameter that is monitored. As recent developments continue to shape the field, significant progress has been made in refining image acquisition techniques to capture high-resolution and comprehensive data for fault detection purposes. In this subsection, the proponents delve into the advancements in image acquisition methods, focusing on the innovative approaches that have emerged to improve the acquisition process.

In [70], the authors used an innovative L-PBF setup with an open cell and a fixed laser to provide quality images for spatter monitoring and enable a large set of data with numerous laser parameters. Two chamber windows enabled laser exposure and the observation of laser–matter interaction. The laser–melt pool interaction could be continuously monitored thanks to this setup. The observations revolved around a high-speed camera (10 kfps frame rate) positioned at 90° because this study focused on spatter analysis. Furthermore, this study used YOLOv4, which shows good performance and application in near real-time conditions in other domains for multi-object detection. By using this method, it showed how the state of the art deep learning object detection techniques outperformed traditional computer vision techniques since the YOLOv4 object detection technique greatly improved recall.

Ref. [66] aimed to address the inability of most existing defect detection methods for 3D monitoring to handle small-sized defects well or detect the defects on the irregular and complex surface effectively. To overcome these difficulties, the authors proposed a local detection strategy that divides the surface points into various groups based on the pluralistic features one by one. The ability to detect defects of various scales and increase the applicability for WAAMed surface is made possible by the combination of individual classification and macro-level analysis of neighborhood characteristics. In order to track the surface quality of the depositing objects in WAAM, the authors developed an in situ 3D laser profilometer inspection (3D-LPI) system based on a laser profilometer. The captured 3D point cloud was mapped to a 2D height topography image to increase processing efficiency, which was confirmed as an efficient solution. Then, using a supervised classifier to divide the pixels into categories of normal and abnormal based on the nearby features, the surface defects were discovered and described.

#### 4.2.2. Novel Algorithms and Methods

Although CNN remains the most commonly used algorithm, there is a trend that shows that fewer papers have made use of it. In 2023, only [68,71,72] made use of CNN, with two of them using it in combination with another algorithm. Only [61] used CNN in 2022. This is most likely due to the proliferation of the use of CNN in the prior years to avoid repeatability and duplication. Thus, researchers and practitioners have explored innovative approaches and novel algorithms to enhance the accuracy, efficiency, and versatility of fault detection systems. In this subsection, novel algorithms are focused on, highlighting the cutting-edge techniques that have emerged as promising solutions for fault detection in AM. These novel algorithms and methods encompass a wide range of methodologies, including deep learning architectures, graph-based models, and hybrid approaches.

According to [75], existing approaches in SLM powder bed monitoring require high-performance systems. However, the industry is prevented from modifying the original machine to develop a setup that is specifically designed for the purpose of acquisition by warranty issues, manufacturer restrictions, or local laws. This work addresses the identified industrial need to put the Digital Image Processing (DIP) for layerwise monitoring into practice without changing the system or impeding production activities at the level of the machine shop floor. In this method, powder bed monitoring includes two approaches: a 2D analysis concerning single layer anomaly detection and 3D volumetric analysis. The former can allow a process intervention during the fabrication. The latter requires the process to be complete and provides a 3D visualization of the object. The performance evaluation highlighted 82.6% and 95.4% for the overall precision and recall, respectively. These values indicate a good result if compared with other methods, especially since a low-cost and embedded system is adopted for monitoring the whole building platform area.

In a recent study by [73], the authors used the Static Thermographic Method (STM) to obtain fatigue life information of scaffold-like structures produced with different printing parameters. Here, static tensile tests were carried out on complete and scaffold specimens using a constant crosshead speed, and an infrared camera was used to track the evolution of the surface temperature. STM is a non-destructive testing technique that uses infrared thermography to measure the surface temperature of a material during a static tensile test. The temperature distribution on the material’s surface is used to assess the material’s fatigue limit. The STM is based on the principle that a material’s surface temperature increases when subjected to a tensile load. This is because the tensile load causes the material to deform, which generates heat. The heat generated is proportional to the stress applied to the material. The advantage of using this approach is that it can severely reduce the testing time to obtain reliable fatigue data for mechanical design.

A novel approach was proposed by [68] that developed two data-driven classification prediction models that use machine learning algorithms to achieve computer-aided defect detection during the FDM AM process. These models monitor sensing signals and interlayer images to predict and classify processing defects, improving product quality and consistency. In this paper, the interlayer surface images and sensing data (vibration signals and infrared temperature) from the FDM process were acquired by arranging a digital microscope and sensors (accelerometer and infrared thermometer) on the printer. Then, two methods of defect classification were tested on the acquired dataset. The first method classifies defects using a transfer learning model based on Swin Transformer and interlayer surface images, and the second method diagnoses defects using a model based on 1DCNN and sensing data. The results showed that the fusion of these two models was more reliable than the prediction using a single ML model. In comparison to the Swin Transformer model and the 1DCNN model alone, the prediction accuracy of the fusion model is more than 8.9% and 9.8% higher. Additionally, the method suggested in this paper can accurately identify the relationship model between printing defects and process parameters, facilitating later online corrections for printing-related defects.

In [65], an algorithm called Inverse Distance Weight K-Nearest Neighbor (IDW-KNN) was proposed to address the imbalance between positive and negative samples. This algorithm can satisfy online detection because it has strong interpretability, fewer parameters, a faster operation speed, and greater flexibility than the commonly employed algorithms. Furthermore, the online detection approach suggested in this paper mines image data to identify relationships between process variables and quality features of interest, enabling real-time process quality monitoring independent of offline analysis. In this work, the image processing algorithm receives the collected images and determines how many of the powder splatters are in a fully melted state. The proposed algorithm can precisely extract the necessary features from a complex background. The quantity of powder spatters is then output to the quality evaluation standard. To enable automatic labeling, the quality corresponding to the powder spatters is divided into four levels based on porosity. In order to address the issue of the imbalanced distribution of quality level samples, the IDW-KNN algorithm is finally proposed. By using the inverse distance method to ensure that the voting weight of the data with a short distance (good quality and normal quality) is high and that of the data with a long distance (slight abnormality and serious abnormality), the IDW-KNN algorithm assigns different weights to four different quality levels based on Euclidean distance, a method that significantly increases the prediction accuracy of the four quality levels.

In [63], the authors developed a semi-supervised clustering-based method to automatically detect spectra patterns that are sensitive to a high density of pores, i.e., the high number of microscopic pores within a unit space. This is equivalent to clustering the spectra into two groups—one relates to high-quality products with a low pores density, and the other relates to low-quality products with a high pores density. Among the existing clustering methods (centroid-based clustering, hierarchical clustering, distribution-based clustering, graph-based clustering), this graph-based clustering method, spectral clustering, is chosen as it shows a good performance when the centroids of different clusters are not separable. Based on the pretrained baseline models, the fundamental idea here is to project an incoming window of spectra as a vector into the eigenspace of spectral clustering and then calculate the distances between the projected vector and the cluster centers of the projected vectors in the high- and low-quality groups, respectively. The quality group whose cluster center is nearer the projected vector is given the same label as the window of spectra.

### 4.3. Future Directions

As the field of image-based fault monitoring in AM continues to evolve, it is crucial to explore and outline the potential future directions for research and development. Building upon the recent advancements discussed earlier, this section delves into the key areas that hold promise for further enhancing fault detection and monitoring in AM processes. Future research endeavors can drive the field towards more robust, efficient, and comprehensive fault monitoring techniques by addressing the existing challenges and gaps in the current state of the art. This section analyzes these potential future directions and discusses their impact on AM quality control, process optimization, and overall manufacturing reliability.

#### 4.3.1. Robust and Accurate Image Processing Algorithms

One of the major obstacles is the need for more robust and precise image processing algorithms. This is due to the complexity of AM processes, the low signal-to-noise ratio (SNR) of AM images, and the high variability of AM components. The complexity of AM processes presents image processing algorithms with unique challenges. AM entails the intricate deposition of materials layer by layer, resulting in complex geometries, surface textures, and internal structures. These variables can introduce various types of defects, such as surface roughness, geometric distortions, porosity, and voids. Advanced image processing techniques are required to detect and differentiate these defects from intentional features or natural variations in AM parts. In addition, AM images frequently have a low signal-to-noise ratio (SNR) because of factors such as lighting conditions, reflections, and image artifacts. Image-based fault detection algorithms can be significantly impacted by noise and interference. Developing robust algorithms that can effectively handle noise and improve signal quality is essential for precise fault detection. A second difficulty is the high variability of AM parts. Each printed component may exhibit unique characteristics and defects, making it challenging to develop image-processing algorithms that are universally applicable. The algorithms must be adaptable and versatile to accommodate various AM processes, materials, and part geometries. They must be capable of learning from diverse datasets and generalizing effectively to unseen or unexpected flaws.

Researchers are examining various algorithm design approaches for image processing to address these obstacles. This includes using sophisticated computer vision techniques such as feature extraction, texture analysis, segmentation, and pattern recognition. Improving the accuracy and robustness of image-based fault detection in AM using machine learning algorithms, such as CNN-like deep learning architectures, has shown promise. Several strategies can be implemented further to advance the current state of image processing algorithms. Advanced feature extraction techniques, such as methods based on deep learning, can detect subtle flaws and variations. Enhancement and noise reduction techniques, such as adaptive filtering and contrast enhancement, can enhance the visibility of flaws. Adaptive and context-aware approaches considering particular AM process characteristics can improve algorithm robustness. Multi-modal data fusion can provide complementary information, and transfer learning can leverage domain-specific knowledge.

#### 4.3.2. Efficient and Scalable Data Acquisition and Analysis Techniques

Further complicating image-based fault detection in AM requires more efficient and scalable data acquisition and analysis techniques. Existing methods for image-based fault detection are frequently expensive and time-consuming. The fault monitoring process can be complex and resource-intensive, requiring specialized imaging systems, cameras, lighting setups, and image-capturing protocols. Acquiring high-quality images with sufficient resolution, clarity, and accuracy is crucial for effective fault detection. However, the cost and complexity associated with the equipment and procedures for data acquisition can pose challenges, particularly when considering large-scale or high-volume AM production scenarios. The need for specialized hardware and controlled imaging environments can limit the scalability and practicality of image-based fault detection methods. These factors may hinder the widespread adoption of such techniques in real-world manufacturing settings. Moreover, analyzing acquired image data is another area that demands attention. The data analysis often involves sophisticated algorithms and computational techniques for image processing, feature extraction, and fault detection. These algorithms require significant computational resources and can be time-consuming, mainly when dealing with large datasets or complex AM parts. The time-consuming nature of data analysis can hinder real-time or near-real-time fault detection in AM. In scenarios where quick feedback and immediate action are necessary, the delays caused by lengthy analysis processes can limit the effectiveness of image-based fault detection methods.

To address these challenges, there is a need for the development of more efficient and scalable data acquisition and analysis techniques. This can involve advancements in hardware, such as designing cost-effective imaging systems that can capture high-quality images efficiently and in real time. Similarly, developing streamlined and optimized algorithms for image processing and fault detection can help reduce computational requirements and speed up the analysis process. Additionally, exploring novel approaches that leverage parallel processing, cloud computing, or edge computing can enhance the scalability and efficiency of data analysis in image-based fault detection. These techniques can distribute the computational workload and enable faster analysis, making real-time or near-real-time fault detection feasible in practical manufacturing environments.

#### 4.3.3. Standardized Methods and Datasets

The development of standardized methods for image-based fault detection is another crucial aspect that requires attention. There currently need to be widely accepted and standardized visual inspection methods for detecting and classifying defects in AM parts. With standardized methods and datasets, reliability, repeatability, and comparability of results across studies and applications are maintained. Evaluating and comparing the performance of various fault detection algorithms, techniques, and systems with standardized procedures and datasets becomes easier. In addition, there is a severe lack of publicly accessible datasets intended for training and assessing fault detection algorithms in AM.

Researchers and practitioners can gain multiple benefits by addressing the lack of datasets. First, having standard datasets tailored for AM fault detection would provide a benchmark for comparing the performance of different algorithms. Researchers could train their models using the same dataset, enabling insightful comparisons. In addition, the availability of publicly accessible datasets would foster collaboration and accelerate the development of new fault detection techniques. Standardized datasets would also enhance the repeatability and reproducibility of experiments by permitting researchers to validate and verify their methods using the same dataset. This would ensure that other researchers’ reported results are accurate and reproducible, thereby enhancing the credibility of the field as a whole. To address the lack of datasets, collaborative efforts are required to generate and curate datasets that represent the diverse range of AM process defects and are accessible to the public. These datasets should consist of different types of materials, printing technologies, and defect characteristics. They should be annotated with accurate ground truth labels for supervised learning approaches.

#### 4.3.4. Lack of Error Detection and Mitigation Research

Although significant progress has been made in image-based fault monitoring for AM, error detection and mitigation research in AM still needs to be improved. Future research should concentrate on developing innovative error detection and mitigation techniques and methods in AM. This would require investigating the root causes of errors, comprehending the underlying mechanisms contributing to their occurrence, and developing effective strategies to detect and mitigate these errors at various stages of the AM process. In addition, it is crucial to encourage and promote the publication of additional research papers that focus on error detection and mitigation in AM. By increasing the number of papers devoted to error detection and mitigation, researchers can share their insights, methodologies, and findings, thereby contributing to a greater understanding of the field’s challenges and potential solutions. This will promote collaboration, knowledge sharing, and the development of innovative techniques that can significantly improve the dependability, efficiency, and quality of AM processes.

## 5. Conclusions

Due to its capacity to produce parts with intricate features, AM is quickly becoming more prevalent in a variety of manufacturing industries. The main advantages of AM are generally design freedom, mass customization, waste reduction, quick prototyping, and the capacity to produce intricate structures. Additionally, compared to conventional manufacturing techniques, AM has more manageable process variables and a more significant interaction between the material’s properties and the process variables. Despite the advantages of AM, it poses some disadvantages such as the formation of faults or defects as a result from the layer-by-layer deposition process. Faults in AM can be categorized according to how it affects the geometry, surface quality, microstructure, or mechanical properties of the by-product. Hence, heavy research has been conducted to monitor faults in AM to ensure the quality and reliability of printed parts. In in-process monitoring, sensors and/or cameras are used to gather data and monitor critical parameters. This paper only focuses on image-based monitoring wherein visual data are captured through a digital camera, IR camera, or another form of camera or vision sensor. Overall, this paper presents a comprehensive survey of image-based fault monitoring in AM. The researchers collected relevant publications from 2019 to 2023, garnering a total of 53 papers. The search statement ““Additive Manufacturing” AND (Fault Detection OR Fault Monitoring OR Defect Detection OR Anomaly Detection) AND (Vision-based OR Image-based)” was used. These papers were then categorized by the type of camera, ML algorithm, and errors detected. An extensive literature review examined the fundamental techniques, methodologies, and algorithms employed in image-based fault detection, highlighting their novelty.

An overview of the critical steps in image-based fault monitoring, such as image acquisition, preprocessing, image analysis, defect identification, real-time monitoring and analysis, and decision making and quality control, was provided. Image acquisition techniques and ML algorithms for fault detection were also investigated. The most commonly used types of camera broadly fall into two categories: optical (digital) and thermographic (thermal or infrared) cameras. However, other cameras and vision sensors have been used such as CMM and scanners. Different algorithms, including CNN, SVM, and KM, were analyzed to demonstrate their efficacy in analyzing AM images and detecting errors. Based on the existing papers, CNN is the most widely used algorithm, with an accuracy of 79.21% to 96.8%, due to its ability to learn spatial hierarchies of features. It also has the ability to learn from invariant features. Another common algorithm used in this area is SVM since it can be used to identify defects in AM parts by classifying images as either “good” or “bad”. Moreover, its accuracy ranges from 60% to 99.7%. Its unique features are its ability to learn non-linear relationships between features and its ability to handle high-dimensional data. Several techniques and methodologies were discussed, highlighting the progress made in this field. These advancements have substantially improved the precision, effectiveness, and scalability of image-based fault monitoring in AM.

This paper explores the significant advancements in image-based fault monitoring in AM by providing an overview of the most significant hardware and software innovations and developments in image-based fault monitoring in AM. These developments have facilitated improved quality control and process optimization by bringing us closer to real-time, automated, and reliable fault detection. There is a trend of CNN being used less in current researches as practitioners are now investigating the use of hybrid and novel approaches in fault detection. Furthermore, the proponents identified several challenges and opportunities for future research, such as the need for more robust image processing algorithms, efficient data acquisition and analysis methods, standardized benchmarks and datasets, and more research in fault monitoring. Overall, this survey highlights the significant advancements made in image-based fault monitoring in AM while identifying the challenges that must be addressed. By pursuing the suggested future directions and addressing the identified challenges, the field of image-based fault monitoring in AM can continue to evolve and contribute to improved quality control, process optimization, and overall manufacturing reliability in the AM industry.

## Figures and Tables

**Table 1:** The seven AM processes, according to ASTM F42, with their advantages, drawbacks, and related AM technologies.

The Seven AM Processes			
AM Process	Advantages	Drawbacks	Related Technologies
Material Jetting	High accuracyLow wasteMultiple material parts and colors under one process	Support often requiredLimited materialsNozzle blockage is commonLow viscosity and strength	NanoParticle Jetting (NPJ)Drop On Demand (DOD)
Binder Jetting	Different colorsHigh range of materialsFast processAllows two materials	Not always suitable for structural parts due to the use of binder materialLong time post-processingHigh porosity, low surface quality	Powder Bed and Inkjet Head (PBIH)Plaster-Based 3D Printing (PB3D)
Vat Photopolymerization	High level of accuracy and good finishAllows transparent materialRelatively quick processTypically, large build areas	Relatively expensiveLong post-processing timeLimited materialsRequires support structures	Stereolithography (SLA)Digital Light Processing (DLP)Continuous Liquid Interface Production (CLIP)Daylight Polymer Printing (DPP)
Powder Bed Fusion	Relatively inexpensiveAbility to integrate technology into small scaleLarge material optionsWide range of materials	Relatively slow speedLack of structural properties in materialsSize limitationsHigh power usageFinish dependent on powder grain sizeThermal stress and degradation is common	Selective Laser Sintering (SLS)Selective Laser Melting (SLM)Electron Beam Melting (EBM)Multi Jet Fusion (MJF)Direct Metal Laser Sintering (DMLS)
Material Extrusion	Widespread, inexpensiveGood material propertiesLow material wasteFairly high fabrication speed	Nozzle radius limitedLow accuracy and speedRequired constant pressure of materialDelamination is common	Fused Deposition Modeling (FDM)Fused Filament Fabrication (FFF)
Energy Deposition	High quality, functional partsSpeed often sacrificed for high accuracy	May require post-processing for desired effectLimited materialThermal stress, requirement for atmosphere control	Laser Engineering Net Shape (LENS),Electron Beam Additive Manufacturing (EBAM)Laser Deposition Modeling (LDM)Wire Arc Additive Manufacturing (WAAM)
Sheet Lamination	High speed, low costEase of material handling	Shrinkage, significant amount of wasteDelamination is common	Laminated Object Manufacturing (LOM)

**Table 2:** List of Publications in Image-Based Fault Monitoring for AM.

Publication Year	Publication Count	References
2019	8	[26,28,29,30,31,32,33,34]
2020	17	[21,35,36,37,38,39,40,41,42,43,44,45,46,47,48,49,50]
2021	11	[51,52,53,54,55,56,57,58,59,60]
2022	8	[61,62,63,64,65,66,67,67]
2023	9	[68,69,70,71,72,73,74,75,76]
Total	53	

**Table 3:** Summary of papers that use image-based monitoring in AM.

Ref	Year	AM Process	Camera Used	ProposedAlgorithm	ErrorsDetected	Accuracy
[67]	2023	FFF	Optical and IR Camera	N/A	Point and Line Defects	N/A
[73]	2023	FDM	Thermographic Camera (FLIR^®^ System model A40)	Static ThermographicMethod	Porosity, Micro delamination, and Micro-cracks	N/A
[33]	2019	L-PBF	Visible-light Camera	Bayesian Classifier	Geometric Errors and Porosity	N/A
[39]	2020	SLM	Machcam 71 MP Camera	CNN	Critical Stripes, Scanning Surface, Upraising Areas, and Recoating Defect	79.21–97%
[50]	2020	N/A	CCD Camera (Lumens DC125)	CNN	Extrusion Speed and Extrusion Temperature	94%
[35]	2020	L-PBF	Thermographic Camera (PYROVIEW 640 G/50 Hz/25° × 19°/compact)	CNN	Splatter and Delamination	96.8%
[51]	2021	FDM	Overhead Webcam	NNGBCSVM	N/A	95% 93% 60%
[75]	2023	SLM	CCD Camera	Digital Image Processing	Powder Bed Spreading	N/A
[36]	2020	FFF	CCD Camera	KM	Porosity and Cracking	N/A
[32]	2019	SLM	24.2 MP Single-Lens Reflex Digital Camera	CNN	Defects Induced by Process Non-conformities (Trace discontinuity, insufficient layer densification, etc.)	99.4%
[70]	2023	L-PBF	High-speed Camera	YOLOv4	Spatter	N/A
[59]	2021	DED	Dual-camera	KNNRFGPOther algorithms (SVM, DT, NB, ANN, AB)	Surface Defects	93.15% 69.86% 67.12%
[72]	2023	PP	Optical Camera (Samsung Galaxy S7)	CNN	Surface Defects	98%
[61]	2022	N/A	Optical Camera	CNN	Bending Deformation in the Printed Concrete Layers	90.5% (in concrete and non-concrete layers) 97.5% (in defected and non-defected layers)
[54]	2021	L-PBF	High-speed Camera	CAE	Melt Pool Defects	95.38%
[41]	2020	N/A	USB Camera (13 MP, FOV 75Degree Autofocus USB Camera with Non-Distortion Lens)	MobileNet-SSD	Surface Defects	N/A
[58]	2021	L-PBF	Area-Scan Hyperspectral Camera	CNN	Surface Roughness	N/A
[74]	2023	FDM	IR Thermocouple	KNN, RF, ANN, Multinomial Logistic Regression, and SVM	Print Regime, Strand Width, Strand Height, and Fusion Ratio	90%
[66]	2022	WAAM	Laser Profilometer Sensor	SVM	Surface Defects	99.8%
[21]	2020	FDM	Optical Camera (Logitech C270)	CNN	Delamination and Predict Warping	Validation: 97.8% Testing: 91%
[26]	2019	FDM	Optical Camera (Logitech C270)	CNN	Under-extrusion and Over-extrusion	98%
[56]	2021	FDM	Optical Camera (Raspberry-pi Camera)	SVMKNNRFDTNB	Warping, Blistering, Porosity, Cracking, Residual Stresses, Poor Surface Finish, Stringing, Material Shrinkage	99.7% 99.4% 97.2% 96.6% 85.9%
[44]	2020	N/A	Webcam	CNN	Filament Tangling (spaghetti-shape errors)	~90%
[37]	2020	TPL	Optical Camera	Seq CNN-LSTM	Part Quality	95.1%
[68]	2023	FDM	Digital microscope (Aomekie USB Microscope) and IR Camera	Swin Transformer algorithm and 1DCNN	N/A	0.979
[57]	2021	FFF	Digital Camera or 3D Scanner	Z-differenceBaggingGBRFL-SVMKNN	Geometrical Defects	0.9737–0.9886 0.9978–0.9992 0.9978–0.9997 0.9978–0.9992 0.9956–0.9959 0.9923–0.9979
[48]	2020	N/A	Optical Camera	R-CNN, SSD, and YOLOv4	Misalignment and Abrasion	N/A
[71]	2023	N/A	Video Camera	Semi-supervised Identification Consistency-based Method	Surface Roughness	25.9–74.1%
[29]	2019	FFF	Optical Camera	3D Model Reconstruction	Layer-by-layer Defects	N/A
[62]	2022	FDM	N/A	Autoencoder and GAN	Warping	N/A
[45]	2020	L-PBF	Multispectral Photodetector Sensor	Graph Theoretic Approach	Porosity	N/A
[69]	2023	FFF	Optical Camera (SVCAM exo264CGE)	N/A	Surface Errors	86.5%
[34]	2019	L-PBF	Visible-light High-speed Camera	SVM	Keyholing Porosity and Balling Instabilities	85.1%
[46]	2020	L-PBF, EB-PBF, BJ	Visible-light Camera, NIR Camera, and MWIR Camera	DSCNN	Surface-visible Defects (recoated blade impacts, binder deposition issues, spatter generation, porosity)	N/A
[28]	2019	N/A	3D Scanning CMM and Computer Vision	DNN	Translation, Scaling Up, Scaling Down, and Rotation	N/A
[38]	2020	FFF	Thermographic Camera	DNN and AI-TSR	Delamination	Delamination Thickness: 95.4% Unacceptable Condition: 98.6%
[60]	2021	N/A	DSLR Camera (Nikon D800E)	CNN	Lack of fusion and Spatter	Same Build: 93.5% Unseen Build: 87.3%
[63]	2022	N/A	Scanning Electron Microscope	Semi-supervised Spectral Clustering Method	Porosity	N/A
[55]	2021	ABS 3D Printing	Thermal Imaging Camera	IRT and CNN	Surface Breaking Holes	90%
[30]	2019	SLS	Thermal Camera	CNN-based Encoder-Decoder Network	Temperature	N/A
[40]	2020	LBAM	Thermal Camera and Pyrometer	CNN and IRNet	Porosity	90%
[53]	2021	WAAM	Vision Sensing System	EPNet and ADRC	Melt Pool Width	94.18%
[52]	2021	SLS	HD Webcam	CNN and Complex TL	Powder Bed Defects	0.958
[65]	2022	N/A	High-speed Camera (5F04M monochrome CMOS high-speed camera)	IDW-KNN	N/A	94–100%
[64]	2022	WAAM	Laser Scanner	ANFISSVRELM	Surface Roughness	N/A
[43]	2020	SLS	Digital Camera	TS-CNN	Warpage, Part Shifting, and Short Feed	94–96%
[76]	2023	FDM	Sony a7 III Camera	TL	Surface Quality	90%
[42]	2020	L-PBF	Infrared Camera	SC with K-SVD	Porosity	N/A
[31]	2019	L-PBF	High-speed Camera	CNN	Porosity	91.2%
[47]	2020	L-PBF	High-speed Camera	Hybrid CNN	Overheating, Irregularity in Process Conditions, and Balling	0.997
[49]	2020	FDM	CMOS Camera	VCSS and FPFH	N/A	N/A

## Data Availability

Not applicable.

## Abbreviations

The following abbreviations are used in this manuscript:

AM	Additive Manufacturing
3DP	3D Printing
RP	Rapid Prototyping
DDM	Direct Digital Manufacturing
SFF	Solid Freeform Fabrication
CAD	Computer-Aided Design
STL	Standard Tessellation Language
ML	Machine Learning
AI	Artificial Intelligence
CCD	Charge-Coupled Device
CMOS	Complementary Metal Oxide Semiconductor
FDM	Fused Deposition Modeling
L-PBF	Laser-Powder Bed Fusion
SLM	Sintering Laser Melting
CNN	Convolutional Neural Network
FFF	Fused Filament Fabrication
GBC	Gaussian Bayes Classifier
NN	Neural Network
SVM	Support Vector Machine
KM	K-Means
DED	Directed Energy Deposition
RF	Random Forest
GP	Gaussian Process
DT	Decision Tree
NB	Naive Bayes
ANN	Artificial Neural Network
AB	AdaBoost
CAE	Convolutional Auto-Encoder
LSTM	Long Short-Term Memory Networks
KNN	K-Nearest Neighbors
EBPBF	Electron Beam Powder Bed Fusion
BJ	Binder Jetting
DSCNN	Dynamic Segmentation Convolutional Neural Network
DNN	Deep Neural Network
AI-TSR	Artificial Intelligence–Thermographic Signal Reconstruction
IRT	Active Infrared Technology
LBAM	Laser-Based Additive Manufacturing
ABS	Acrylonitrile Butadiene Styrene
ADRC	Active Disturbance Rejection Control
WAAM	Wire Arc Additive Manufacturing
TL	Transfer Learning
ANFIS	Adaptive Neuro-Fuzzy Inference System
SVR	Support Vector Regression
VCSS	Voxel Cloud Connectivity Segmentation
FPFH	Fast Point Feature Histogram
CMM	Coordinate-Measuring Machine
STM	Static Thermographic Method
GAN	General Adversarial Network
